# Structural alteration of the endothelial glycocalyx: contribution of the actin cytoskeleton

**DOI:** 10.1007/s10237-017-0950-2

**Published:** 2017-08-14

**Authors:** Weiqi Li, Wen Wang

**Affiliations:** 1 Institute of Bioengineering and School of Engineering and Materials Science, Queen Mary University of London, London, E1 4NS UK; 2 Department of Laboratory Medicine, Nanfang Hospital, Southern Medical University, Guangzhou, 510515 People’s Republic of China

**Keywords:** Endothelial cell, The glycocalyx, Laminar shear stress, Actin depolymerisation

## Abstract

The endothelial glycocalyx is a carbohydrate–protein layer that lines the luminal surface of the endothelium. It anchors to the cell membrane via its core proteins that share extended link to the actin cytoskeleton. It is widely accepted that those protein domains and the attached carbohydrates are susceptible to pathological changes. It is unclear, however, to what extent the actin cytoskeleton contributes to the glycocalyx stability. In this study, we investigate the role of the actin cytoskeleton in the maintenance of the glycocalyx under static and laminar flow conditions in vitro. Our results show that in the static culture medium neither rapid actin depolymerisation nor prolonged actin disturbance leads to glycocalyx disruption from the apical surface of human umbilical vein endothelial cells. However, when endothelial cells are exposed to laminar flow for 24 h, the glycocalyx is seen to shift to the downstream peripheral region of the cell surface. The mean fluorescence intensity decreases to $91.9 \pm 2.5\%$ of the control. When actin depolymerisation is introduced, the intensity decreases significantly to $54.7 \pm 1.3\%$, indicating a severe disruption of the glycocalyx. Similar changes are observed in human aortic endothelial cells, where the intensity of the glycocalyx is reduced to $72.8 \pm 1.6\%$ of the control. Collectively, we demonstrate that the actin cytoskeleton contributes to structural stability of the glycocalyx under shear stress. Our results can be used to develop new strategies to prevent shedding of the glycocalyx in cardiovascular diseases.

## Introduction

The luminal surface of the vascular endothelium is covered by a brush-like layer of membrane-bound carbohydrate-rich molecules. This layer is between a few hundred nanometres and a few micrometres in thickness and is known as the endothelial glycocalyx (Tarbell et al. 2014). The glycocalyx is essential for modulating a number of vascular events, for example, vascular permeation (Michel and Curry 1999; van Haaren et al. 2005; Vink and Duling 2000), inflammatory response (Constantinescu et al. 2003; Mulivor and Lipowsky 2002) and vasodilatation (Pahakis et al. 2007). Damage to the glycocalyx layer leads to endothelial dysfunction and thereby contributes to the development and progression of cardiovascular diseases such as atherosclerosis and renal diseases (Reitsma et al. 2007). Advance in knowledge of the stabilisation of the glycocalyx, therefore, is of great importance.

The glycocalyx network comprises glycoproteins and proteoglycans. The glycoproteins are characterised by short, carbohydrate side chains capped with sialic acid, while the proteoglycans are decorated with long, unbranched glycosaminoglycans (GAGs, including heparan sulphate, chondroitin sulphate and hyaluronan). Both of them consist of core proteins to which one or more sugar side chains are covalently anchored (Reitsma et al. 2007). The intactness of the glycocalyx can be measured by its thickness, intensity or coverage, depending on the techniques used to visualise the glycocalyx. For example, thickness estimation is commonly used in electron microscopy studies, while measurement of intensity or coverage is preferable when fluorescence imaging, e.g., confocal microscopy, is employed (Reitsma et al. 2007; Tarbell et al. 2014).

While many studies reveal the environmental factors that fragment or preserve the glycocalyx (Becker et al. 2015), only a handful of studies shed light on structural stabilisation of the glycocalyx. Previous research drew attention to the interaction of the glycocalyx components but the resulting findings found that enzymatic removal of individual GAG alone did not collapse the remaining structure (Zeng et al. 2012). Structural connection between the glycocalyx and the actin cytoskeleton has not been recognised until the ultrastructure of the glycocalyx was demonstrated in Squire et al. (2001), when Michel and co-workers proposed that the glycocalyx anchors to the actin cytoskeleton via a hexagonal arrangement. On the basis of this model, Weinbaum et al. (2003) further suggested that it may act as a mechanotransducer to convert shear stress to the actin cytoskeleton. If the integrity of the glycocalyx is compromised, the adaptation of the actin cytoskeleton to shear stress is suppressed (Thi et al. 2004), leading to the failure of cell migration, elongation and alignment along the shear stress vector (Ebong et al. 2014; Moon et al. 2005; Yao et al. 2007). Since then, the role of the glycocalyx in actin reorganisation has been widely acknowledged, however, whether the actin cytoskeleton induces a reciprocal effect to the glycocalyx remains poorly understood. In addition, like the glycocalyx, the actin cytoskeleton is susceptible to the mediators of inflammation and oxidant substances (Gruenheid and Finlay 2003; Moldovan et al. 2006). The latter would trigger aberrant actin depolymerisation in endothelial cells. Exploring the relationship between the actin cytoskeleton and the glycocalyx offers an alternative to understand the stability of the glycocalyx in diseased conditions and helps to develop new approaches to preserve the glycocalyx.

In the present study, we aim to investigate the contribution of the actin cytoskeleton to the structural stability of the glycocalyx in vitro. Specifically, we establish two states of actin depolymerisation with the use of cytochalasin D (CD). One is rapid depolymerisation which quickly disrupts the actin using a high concentration of CD, and the other is prolonged actin disruption. The latter conditions employ a low CD concentration to persistently depolymerise the actin cytoskeleton and are used for studying the long-term effect. The glycocalyx intactness is quantified under both conditions, and the effect of prolonged actin depolymerisation is further examined under shear stimulation.

## Materials and methods

### Cell culture

Human umbilical vein endothelial cells (HUVECs) and human aortic endothelial cells (HAECs) were purchased from Lonza (Slough, UK) and grown in medium 199 (Invitrogen, Paisley, UK) supplemented with foetal bovine serum (10%), $\upbeta$-endothelial cell growth factor (1 ng/ml), bovine neural extract ($3~\upmu \hbox {g}/\hbox {ml}$), thymidine ($1.25~\upmu \hbox {g}/\hbox {ml}$), heparin (10 U/ml), penicillin (100 U/ml) and streptomycin ($100~\upmu \hbox {g}/\hbox {ml}$). All supplements were purchased form Sigma. Before experiment, endothelial cells were collected by incubating with trypsin-EDTA solution (0.5%, Sigma, Dorset, UK) for 5 min and then plated on a glass, non-coating coverslip at a density of $2500~\hbox {cells}/\hbox {cm}^{2}$ (HUVECs) and $6000~\hbox {cells}/\hbox {cm}^{2}$ (HAECs). They were further cultured for a week, allowing full recovery of the glycocalyx on the cell surface (here defined as an intensive, continuous FITC-WGA binding layer) (Bai and Wang 2012).

### Cytochalasin D (CD) treatment

The role of the actin cytoskeleton was investigated using CD (Sigma). Its dosage and duration for HUVECs were determined according to a serial dilution from 2000 to 30 nM. Specifically, we incubated confluent HUVECs with 1000 nM CD for 10 min in order to attain rapid actin depolymerisation without cell detachment. For prolonged depolymerisation, the cells were exposed to 250 nM CD for 24 h. In shear stress study, HUVECs were pre-incubated with 250 nM CD for 1 h and then subjected to shear flow concurrent with 30 nM CD, thus maintaining cells in their depolymerised state throughout the flow experiment. Since HAECs were more susceptible to CD, CD concentration was reduced to 100 nM to generate prolonged actin depolymerisation without compromising the cell attachment. The degree of actin depolymerisation was determined by evaluating actin filament number (which is described in the quantification session) and cell retraction. The latter was measured using projected cell area obtained from bright field images.

### Shear stress application

Shear stress apparatus described previously was adapted in our study (Bai and Wang 2014; Yao et al. 2007). Flow chamber was manufactured using parallel plates, with one plate built with input and outlet connectors, while the other cut with a rectangular groove. A coverslip containing endothelial cell monolayer was fitted into this groove and remains level with the plate surface. Fluid delivery system was set up as a closed loop using two reservoirs and a peristaltic pump [details can be found in reference Bai and Wang (2014)]. Circulating medium was prepared using medium 199 plus all supplements as described above. All flow experiments were conducted at laminar shear stress of $15~\hbox {dyn}/\hbox {cm}^{2}$.

### Immunofluorescence staining

FITC-wheat germ agglutinin (FITC-WGA, Sigma) was used to label the glycocalyx as it binds primarily to sialic acid and *N*-acetyl-D-glucosamine, which are common residues for glycoproteins and proteoglycans, respectively. Usage of WGA provided an overview of structural changes in the glycocalyx (Barker et al. 2004; Haldenby et al. 1994; Reitsma 2011). Cell tracer far-red (CTFR, Invitrogen) or cell tracker red (CTR, Invitrogen) was used to display the cytoplasm of a cell (both pseudocolorised in magenta). Endothelial cells were fixed in 4% paraformaldehyde in PBS for 8 min and then incubated with FITC-WGA ($10~\upmu \hbox {g}/\hbox {ml}$) and CTFR or CTR ($10~\upmu \hbox {M}$) simultaneously at room temperature for 15 min. As such, FITC-WGA was selectively bound to the glycocalyx components on the apical cell surface. Over-dose and over-time incubation of FITC-WGA leaded to dye diffusion into the cytoplasm. The labelled samples were immediately used for confocal scanning. For visualisation of the actin cytoskeleton, the fixed cells were permeabilised with 0.1% Triton X-100 (Sigma) for 10 min, blocked with 1% BSA (Sigma) for 30 min and subsequently incubated with $10~\upmu \hbox {g}/\hbox {ml}$ rhodamine-phalloidin (Sigma) in 1% BSA for 30 min. These cells were finally counterstained with DAPI (Sigma), mounted in ProLong$^{{\textregistered }}$ Gold reagent (without DAPI, Invitrogen) and scanned by confocal microscopy within 1 week.

### Image acquisition and analysis

#### Image acquisition

All fluorescence images (8 bit) were acquired using a Leica TCS SP2 confocal laser scanning microscopy with a Leica HCX PL APO Lbd.BL $63\times /1.4$ oil objective. The field of view was captured with a pixel format of $1024 \times 1024$, which in turn created a pixel size of $232.5~\hbox {nm} \times 232.5~\hbox {nm}$. The images were scanned from basal cell surface to apical cell surface at an interval of $0.3~\upmu \hbox {m}$. Laser power, gain and offset value were optimised to achieve optimal brightness and to avoid photobleaching. To prevent convolution between two different fluorophores, sequential scanning mode was employed.

#### Glycocalyx quantification

*Z*-stack images were presented using maximum-intensity projection in Image J (NIH). Images were thresholded above the background intensity level and below the saturated level of 250–255 to exclude junctional regions. The boundary of each cell was outlined according to the labelling of FITC-WGA. The intactness of the glycocalyx on the entire apical surface of each cell was determined by reading the mean fluorescence intensity (MFI) (Bai and Wang 2014). MFI obtained from different groups was normalised to the parallel control for comparison. To quantify the distribution of the glycocalyx under flow conditions, a straight line was drawn perpendicularly to the flow direction cutting across the centroid of cell nucleus, dividing a cell into upstream and downstream regions. MFI on each side was measured and directly used for comparison.

#### F-actin quantification

The actin cytoskeleton, in the form of either bundles or networks, was fundamentally a filamentous structure. It was measured using FilaQuant, an image processing tool developed for measuring ridges and filaments (Birkholz et al. 2010; Matschegewski et al. 2012). An actin filament usually appeared as a curve with more than one segment. Every line segment was tracked and joined to form a consecutive filament. The number of these consecutive filaments per cell was used to represent the degree of actin depolymerisation. In some cases, the filaments tracked at the junctional areas were overlap by neighbour cells and difficult to be separated. Those filaments were excluded from counting.

### Statistical analysis

Data were presented as mean ± s.e.m of five independent experiments. Statistical analysis was performed in SPSS 24 using two-tailed independent samples *t* test and one-way ANOVA with Bonferroni or Dunnett’s T3 for multiple means comparison (depending on Levene’s statistic for homogeneity of variance). Difference was considered significant if $P < 0.05$.

## Results

### The endothelial glycocalyx is preserved during rapid actin depolymerisation and subsequent repolymerisation

**Fig. 1:**
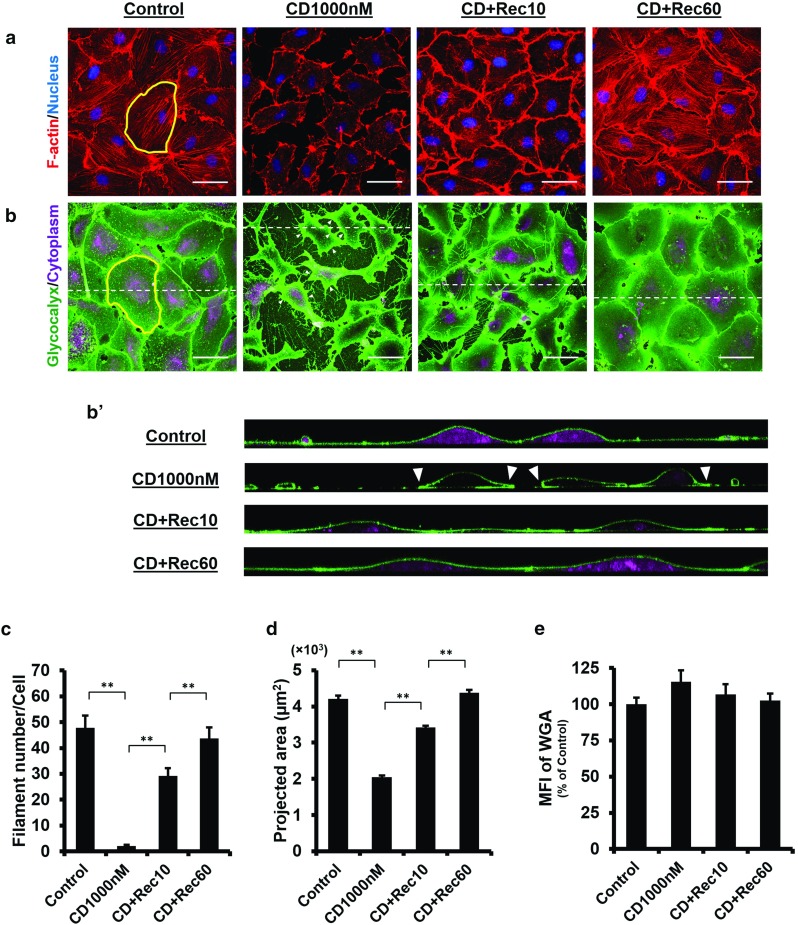
Endothelial glycocalyx is preserved during rapid actin depolymerisation and subsequent repolymerisation. Confluent HUVECs were incubated in medium supplemented with 1000 nM cytochalasin D (CD) for 10 min. $\hbox {CD}+\hbox {Rec}10$: CD plus 10 min recovery, $\hbox {CD}+\hbox {Rec}60$: CD plus 60 min recovery. **a** Immunofluorescence images show that the actin cytoskeleton is depolymerised with CD treatment, followed by partial recovery at 10 min and fully repolymerisation at 60 min in fresh CD-free medium. **b** WGA staining is maintained over the cell surface during the rapid actin depolymerisation and the subsequent repolymerisation. In particular, under rapid depolymerisation, cells retract severely, leading to significant folding of the membrane protrusion and concentrated WGA at this region (as indicated by *triangle*). $\mathbf{b}^{\prime }$ $x{-}z$ cross-sectional images correspond to the *dash lines* drawn in the $x{-}y$ planes. The WGA layer on *top* of the apical cell surface remains continuous. ${ Scale~bar}= 50~\upmu \hbox {m}$. The actin cytoskeleton and WGA are quantified over a whole cell as outlined in *yellow*. **c** Changes in the number of actin filaments per cell. **$P<0.01$ by ANOVA with Dunnett’s T3. **d** Changes in the projected cell area during the rapid actin depolymerisation and the subsequent repolymerisation. **$P<0.01$ by ANOVA with Dunnett’s T3. **e** The mean fluorescence intensity (MFI) of the WGA on the cell surface (junctional regions excluded) remains largely unchanged, which is consistent with results in **b**

Following our previous study on the spatial and temporal development of the glycocalyx in vitro, we investigated the contribution of the actin cytoskeleton to the stability of the glycocalyx using cytoskeletal destabilised agent, cytochalasin D (CD) in the present study. Without CD treatment, the actin in human umbilical vein endothelial cells (HUVECs) was present as filaments across the cell centre and/or along the cell periphery. After 10 min 1000 nM CD incubation, the filament structure was abolished (Fig. 1a). The cells retracted, leaving few contacts to their neighbours. With the increase in CD concentration (e.g., 2000 nM) and/or incubation time, the cells were seen to detach from the glass coverslip (results under such conditions were not shown). The degree of actin depolymerisation induced by CD was quantified by examining the actin filament number and the projected cell area. The filament number per cell was $47.8 \pm 4.8$ prior to CD treatment and the value dropped to $2.0 \pm 0.5$ at 1000 nM CD ($P<0.0001$; Fig. 1c). The change in the projected area (Control $4214~\pm ~90~\upmu \hbox {m}^{2}$ vs. CD $2044~\pm ~54~\upmu \hbox {m}^{2}$, $P<0.0001$; Fig. 1d) was consistent with the reduction in the filament number, indicating that the actin cytoskeleton was depolymerised. Interestingly, if we compare the glycocalyx layer on actin-depolymerised cells to that of the control, it was seen that the glycocalyx distributed continuously on the entire cell surface, with a more concentrated pattern at the protrusion region. Actin depolymerisation did not disrupt this layer (Fig. 1b). The mean fluorescence intensity (MFI) of the glycocalyx increased slightly but the change remained insignificant with respect to the control (CD $115.4 \pm 7.9\%$ vs. Control, $P=0.100$, Fig. 1e). The glycocalyx remained stable even though the actin cytoskeleton was rapidly depolymerised in static culture medium.

In addition to links between the actin cytoskeleton and the glycocalyx, the actin cytoskeleton also regulates plasma membrane tension, which in turn could influence molecules anchoring on the membrane. Stability of the glycocalyx was, therefore, also examined during the process of actin repolymerisation. Following the replacement of CD-free medium, the actin cytoskeleton was seen to repolymerise within 10 min ($\hbox {CD}+10\hbox {Rec}$ $29.2 \pm 3.1$ vs. CD $2.0 \pm 0.5$, $P<0.0001$; Fig. 1c). Although the actin repolymerisation was observed over the entire cell, the staining was much more intensive at the junctional regions (Fig. 1a), suggesting that actin repolymerisation initiated from the periphery to the centroid of the cell. After 60 min, actin filaments recovered to the pre-treatment level ($\hbox {CD}+60\hbox {Rec}$ $43.7 \pm 4.3$ vs. Control $47.8 \pm 4.8$, $P=0.528$; Fig. 1c). In parallel to actin filament recovery, cells started spreading at 10 min ($\hbox {CD}+10\hbox {Rec}$ $3418\pm 48~\upmu \hbox {m}^{2}$ vs. CD $2044\pm 54~\upmu \hbox {m}^{2}$, $P<0.0001$; Fig. 1d) until the actin cytoskeleton was fully repolymerised. The final projected area, at $4382 \pm 74~\upmu \hbox {m}^{2}$, was comparable to the control ($P=0.146$; Fig. 1d). These results demonstrated that the disruption of the actin cytoskeleton was reversible. Interestingly, a continuous glycocalyx layer was observed on the cell surface throughout the repolymerisation process (Fig. 1b). There was no significant difference in MFI between recovery groups and the control ($P=0.284$, Fig. 1e), indicating that the glycocalyx was preserved even though the membrane tension was fluctuated during the process of actin depolymerisation and subsequent repolymerisation in static culture medium.

### The endothelial glycocalyx is preserved after prolonged actin depolymerisation

**Fig. 2:**
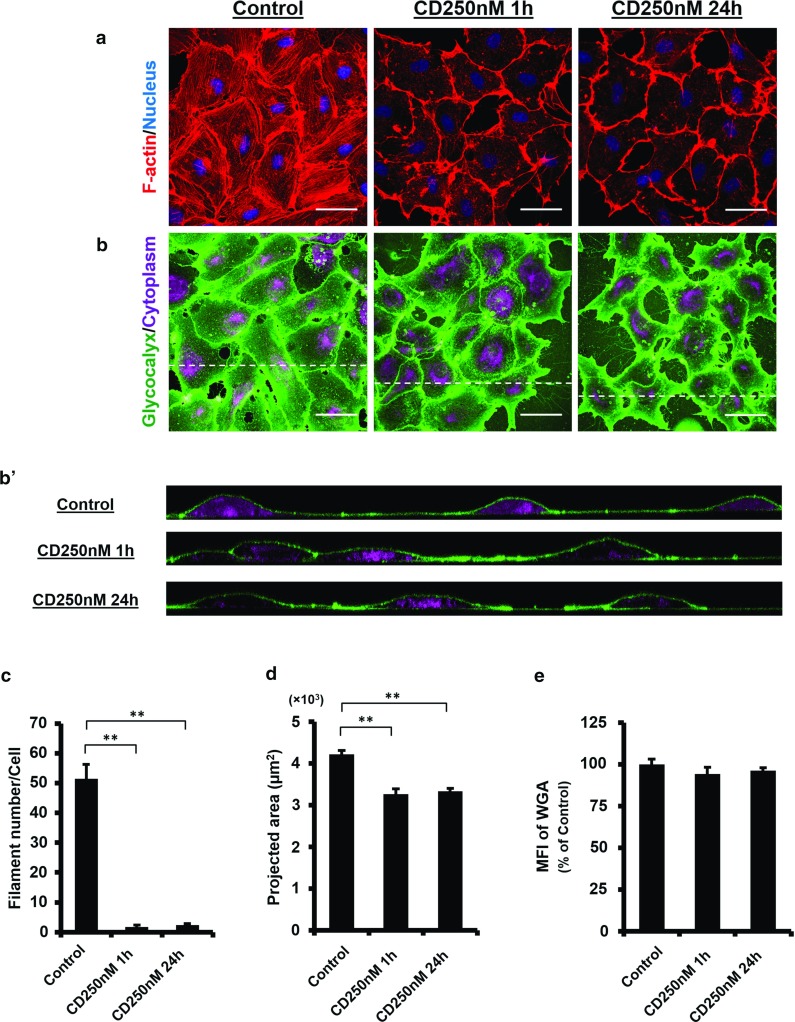
Endothelial glycocalyx is preserved following prolonged actin depolymerisation. Confluent HUVECs were exposed to 250 nM CD for 1 and 24 h. **a** Stack images show that the actin cytoskeleton across the entire cell is completely depolymerised following prolonged CD treatment. **b** and $\mathbf{b}^{\prime }$ The WGA layer is well preserved. ${ Scale~bar}= 50~\upmu \hbox {m}$. **c** and **d** Actin filament number per cell and the average cell area. **$P<0.01$ by ANOVA with Dunnett’s T3. **e** MFI of the WGA

CD concentration at 1000 nM induces rapid actin depolymerisation but may leave insufficient time for the glycocalyx to respond. At this high CD concentration, cell detachment occurs at prolonged incubation time, making it difficult to evaluate the long-term effect of actin depolymerisation on the glycocalyx layer. In order to address this issue, we reduced the CD concentration to 250 nM and maintained actin depolymerisation for 24 h. As expected, the actin cytoskeleton (except those at the junctional regions) were completely lost after 1-h CD treatment and remained depolymerised at 24 h (Fig. 2a). The filament number was reduced to $1.8 \pm 0.5$ per cell at 1 h and to $2.4 \pm 0.5$ at 24 h (vs. Control, $P<0.0001$; Fig. 2c). Although the projected cell area at 1 and 24 h decreased to $3268 \pm 116~\upmu \hbox {m}^{2}$ and $3336\pm 61~\upmu \hbox {m}^{2}$, respectively (Fig. 2d), the reductions were much smaller than that observed at 1000 nM concentration (vs. CD 1000 nM $2044 \pm 54~\upmu \hbox {m}^{2}$, $P<0.0001$). Cell spreading was largely sustained under such condition. The glycocalyx layer remained dense and uniform over the cell surface (Fig. 2b). This was further confirmed by MFI study, where insignificant change was seen to the control (CD 250 nM 24 h $96.2 \pm 1.7\%$ vs. Control, $P=0.288$; Fig. 2e).

Together with results obtained from the rapid actin depolymerisation, we demonstrated that under static condition, the glycocalyx layer on the cell surface remained largely intact independent of either rapid or sustained actin cytoskeleton disruption.

**Fig. 3:**
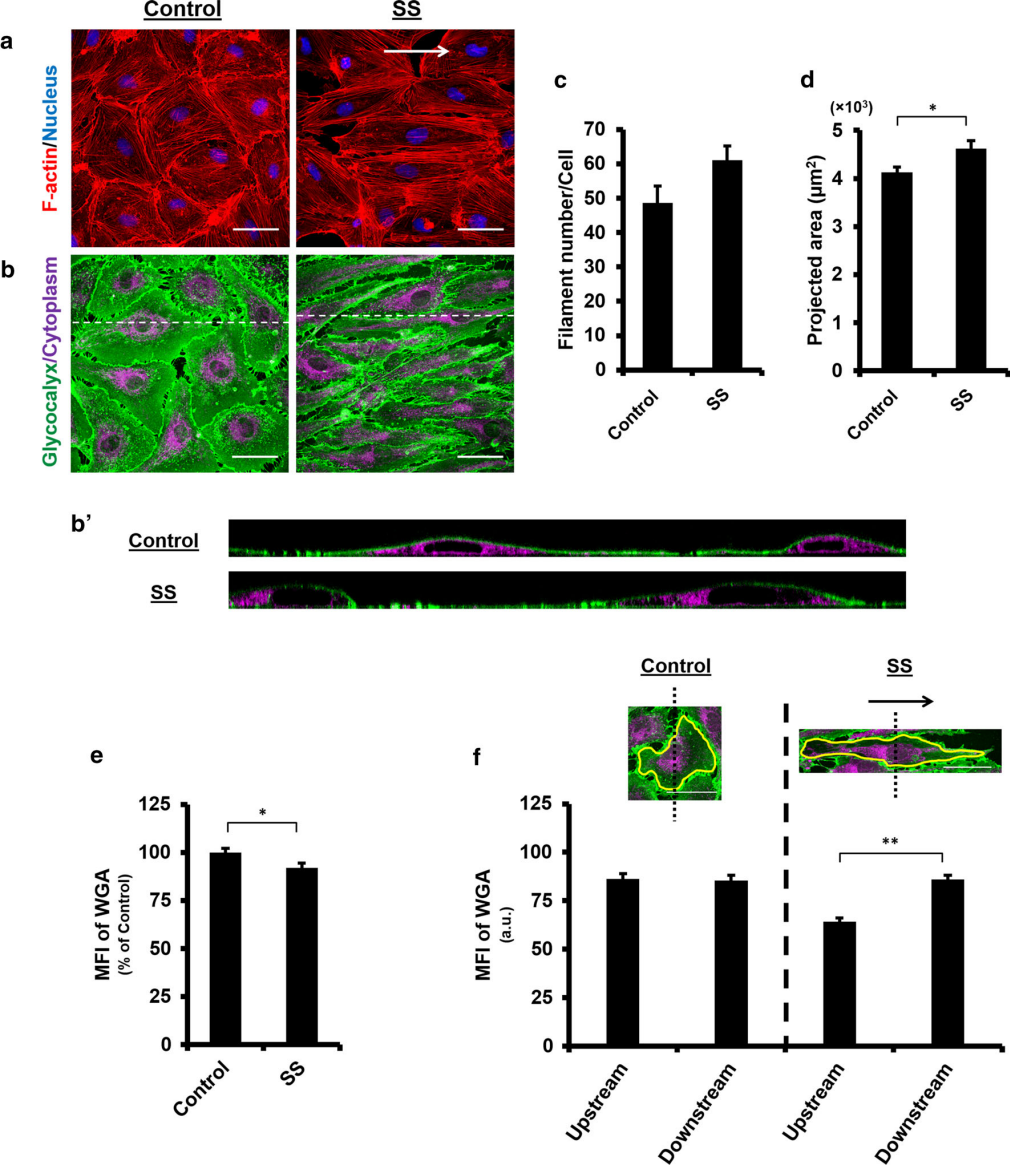
Redistribution of the glycocalyx to the downstream of the cell surface under laminar shear stress. Confluent HUVECs were subjected to a nominal shear stress (SS) of $15~\hbox {dyn}/\hbox {cm}^{2}$ for 24 h. *Arrow* indicates flow direction. **a** The actin cytoskeleton is reorganised under shear flow stimulation, leading to cell alignment parallel to the flow direction. **b** and $\mathbf{b}^{\prime }$ The WGA layer is maintained and seen to shift to the downstream of the cell surface under shear stress. ${ Scale~bar}= 50~\upmu \hbox {m}$. **c** Actin filament number per cell increases under the shear stress, although the increase is not statistically significant which may be due to the formation of bigger stress fibres. **d** Projected cell area increases under SS. *$P<0.05$ by independent *t* test. **e** MFI of the WGA decreases under SS, indicating the WGA layer is partially compromised when exposed to SS. *$P<0.05$ by independent *t* test. **f** Upstream and downstream of a cell are divided by a *dotted line* across nucleus centre. MFI of the WGA becomes significantly larger at the downstream side of the cell under SS. **$P<0.01$ by independent *t* test

### Redistribution of the glycocalyx to the downstream of the cell surface under laminar shear stress

Having demonstrated the impact of actin disruption on the glycocalyx under static condition, we next evaluated the actin contribution in the presence of shear flow. By exposing HUVECs monolayer to $15~\hbox {dyn}/\hbox {cm}^{2}$ laminar shear stress for 24 h, we found that actin filaments mainly distributed on the cell periphery and realigned parallel to the flow direction (Fig. 3a), leading to remarkable cell elongation and reorientation parallel to the flow direction. The filament number increased slightly (SS $61.0 \pm 4.2$ vs. Control, $P=0.060$; Fig. 3c), and the cells became larger, with an average projected area of $4619 \pm 162~\upmu \hbox {m}^{2}$ (vs. Control, $P=0.013$; Fig. 3d). The MFI of the glycocalyx decreased ($91.9 \pm 2.5\%$ vs. Control, $P=0.017$; Fig. 3e), albeit not to a great extent. Notably, the glycocalyx on elongated cells was seen to accumulate at the downstream region (Fig. 3b). This pattern of redistribution was further examined by dividing cell surface into the upstream and downstream areas using a straight line drawn perpendicular to the flow direction across the nucleus centroid. Of the Control group, the MFI measured at the two regions were comparable ($P=0.797$, Fig. 3f), whereas with shear stress stimulation, the MFI at the downstream area was significantly larger than that at the upstream area ($P<0.0001$, Fig. 3f). These results denoted that the glycocalyx reorganised in response to the shear stress, with a greater distribution on the downstream surface of the cells.

**Fig. 4:**
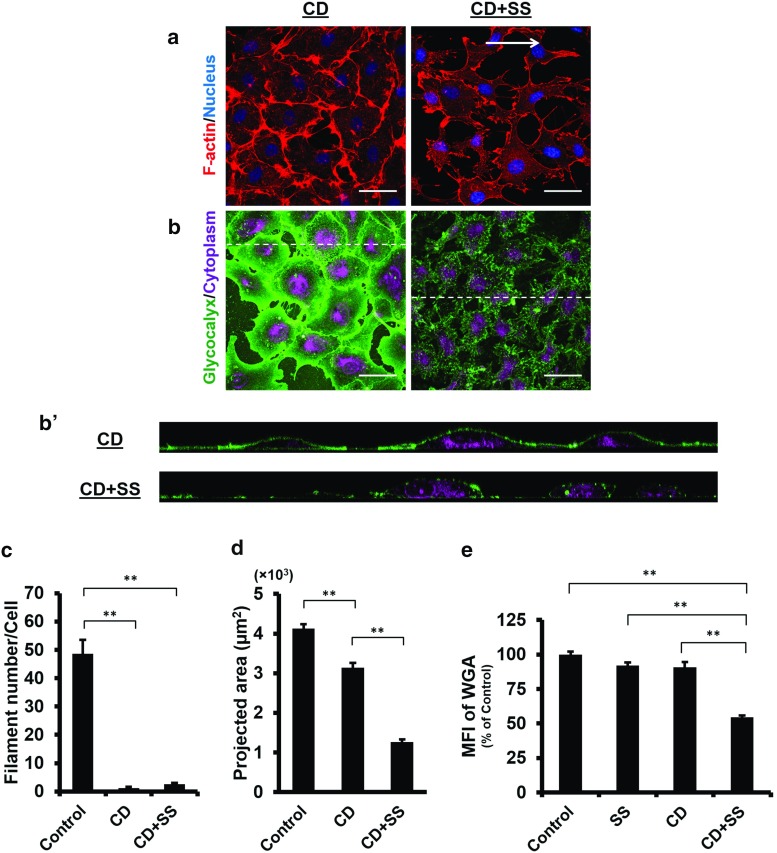
Endothelial glycocalyx on actin-depolymerised cells is severely disrupted in the presence of the laminar shear stress. Confluent HUVECs were pre-treated with 250 nM CD for 1 h and subsequently subjected to a nominal SS of $15~\hbox {dyn}/\hbox {cm}^{2}$ concurrently with 30 nM CD for 24 h. Control: without treatment. CD: CD treatment alone, CD+SS: CD treatment plus SS exposure. *Arrow* indicates flow direction. **a** The actin cytoskeleton is depolymerised in the presence of CD, as the result cells do not realign in response to SS. **b** and $\mathbf{b}^{\prime }$ In actin-depolymerised state, the WGA layer on the cell surface observed in static medium is disrupted severely by SS, leaving only a few patches on the cell surface. ${ Scale~bar}= 50~\upmu \hbox {m}$. **c** Actin filament number is reduced significantly in comparison with the Control. **$P<0.01$ by ANOVA with Dunnett’s T3. **d** Cell area decreases significantly following CD treatment. Under SS, further significant reduction in the cell area is observed. **$P<0.01$ by ANOVA with Dunnett’s T3. **e** MFI of the WGA shows significant reduction in the glycocalyx on actin-depolymerised cells under SS. **$P<0.01$ by ANOVA with Dunnett’s T3

### The glycocalyx on actin-depolymerised cells is severely disrupted in the presence of shear stress

The stability of the glycocalyx layer on actin-depolymerised cells was further investigated under the same flow conditions as described above. As we noted in actin depolymerisation studies, HUVECs partially retracted when exposed to 250 nM CD and detached when shear flow was concurrently applied. To maintain cell attachment under such condition, cells were pre-treated with 250 nM CD for 1 h in order to create an actin-disrupted state and subsequently subjected to circulating medium with a lower concentration of CD, i.e., 30 nM. As shown in Fig. 4a, the actin cytoskeleton was almost completely disrupted at the end of shear stress stimulation ($\hbox {CD}+\hbox {SS}~2.6 \pm 0.5$ vs. Control, $P<0.0001$; Fig. 4c). Due to the loss of the actin cytoskeleton and its reorganisation, cells not only failed to elongate and realign to the flow direction, but also retracted more considerably than those without shear stress exposure ($\hbox {CD}+\hbox {SS}~1268 \pm 64~\upmu \hbox {m}^{2}$ vs. CD $3136 \pm 129~\upmu \hbox {m}^{2}$, $P<0.0001$; Fig. 4d). More importantly, on those actin-depolymerised cells, the glycocalyx was observed to shed severely, leaving only a few patches on the cell surface (Fig. 4b). The MFI of the glycocalyx decreased by 40% compared to other groups ($\hbox {CD}+\hbox {SS}$ $54.7 \pm 1.3\%$ vs. Control, $P<0.0001$; Fig. 4e). These results demonstrated that the actin cytoskeleton was essential for the stabilisation of the glycocalyx under flow conditions.

**Fig. 5:**
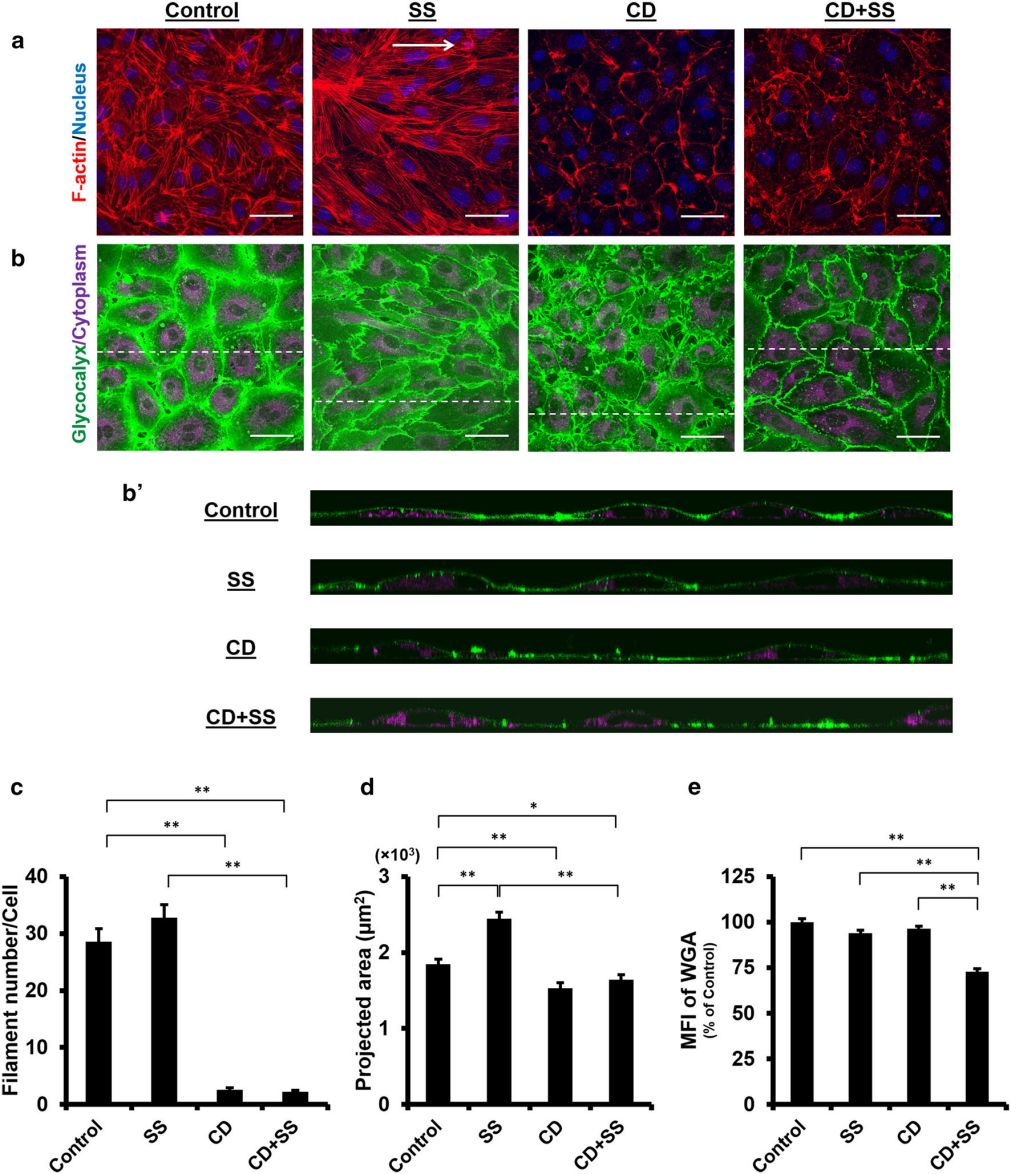
Glycocalyx on actin-depolymerised HAECs is compromised under laminar flow condition. Confluent HAECs were pre-treated with 100 nM CD for 1 h and subsequently subjected to a nominal SS of $15~\hbox {dyn}/\hbox {cm}^{2}$ concurrently with 30 nM CD for 24 h. Control: static culture. SS: shear stress exposure alone, CD: CD treatment alone, $\hbox {CD}+\hbox {SS}$: CD treatment plus SS exposure. *Arrow* indicates flow direction. **a** The actin cytoskeleton is reorganised in response to SS; however, this phenomena is completely abolished in the presence of CD, leading to cells remaining cobblestone-like morphology under SS. **b** and $\mathbf{b}^{\prime }$ The well preserved WGA layer under SS is disrupted in the concurrence of CD. ${ Scale~bar} = 50~\upmu \hbox {m}$. **c** Regardless of SS, actin filament number drops in the presence of CD. **$P<0.01$ by ANOVA with Dunnett’s T3. **d** Projected cell area increases after SS stimulation, whereas it remains unchanged when CD is concurrently applied. *$P<0.05$, **$P<0.01$ by ANOVA with Dunnett’s T3. **e** MFI of the WGA on actin-disrupted cells is reduced under SS. **$P<0.01$ by ANOVA with Dunnett’s T3

Furthermore, we have studied effects of actin depolymerisation on the glycocalyx using a different cell type, human aortic endothelial cells (HAECs). As with HUVECs, actin-depolymerised state of HAECs was sustained over 24 h, the filament number decreased from $28.9 \pm 2.3$ (Control) to $2.6 \pm 0.3$ (CD treatment alone) and to $2.3 \pm 0.3$ (CD treatment and SS exposure). This represented a significant change in comparison with the Control ($P<0.0001$, Fig. 5c). The reduction in the projected area was much smaller than that found on HUVECs, albeit the difference was still significant, i.e., $P=0.002$ (CD $1533 \pm 71~\upmu \hbox {m}^{2}$ vs. Control $1842 \pm 70~\upmu \hbox {m}^{2})$ and $P=0.038$ ($\hbox {CD}+\hbox {SS}$ $1644 \pm 64~\upmu \hbox {m}^{2}$ vs. Control; Fig. 5d). Similar changes in the MFI of the glycocalyx were observed. The MFI obtained under SS was maintained at $93.9 \pm 1.5\%$ of the Control ($P=0.056$, SS vs. Control, Fig. 5e), whereas the MFI was reduced to $72.8 \pm 1.6\%$ of the Control in the concurrence of CD ($P<0.0001$, $\hbox {CD}+\hbox {SS}$ vs. Control, Fig. 5e), confirming that the actin cytoskeleton plays an important role in maintaining the glycocalyx stability under shear condition.

## Discussion

The endothelial glycocalyx is the carbohydrate–protein complex on luminal surface of the endothelium. It anchors via its core proteins to the actin cytoskeleton. In order to identify the role of the actin cytoskeleton on the stability of the glycocalyx, we established two states of actin disruption using CD. One was the rapid depolymerisation, where a high concentration of CD was used for short-term incubation and the other was the prolonged disruption, in which a low concentration of CD was applied over a prolonged time to study the long-term effect of actin depolymerisation on the glycocalyx.

Without the support of the actin cytoskeleton, cell retracted. The extent of cell retraction depended on the CD concentration. Under rapid actin depolymerisation, the high CD concentration resulted in significant decreases in the projected cell area, whereas the low concentration of CD in the prolonged incubation only caused partial cell shrinkage. For the latter, actin staining at the junctional regions of the cell remained intensive, indicating that actins at these regions still existed. Since adherens junctions share the characteristic of binding cells through actins (Hoelzle and Svitkina 2012), the staining pattern suggested that adherens junctions were sustained in the prolonged actin depolymerisation. The remaining actins at the junctional area and the associated adherens junctions functioned to restrain cells from significant retraction. Despite this, our quantification data confirmed complete actin disruption across the cytoplasm and protrusion under both conditions. The glycocalyx layer on the cells was preserved following either rapid or prolonged actin depolymerisation, suggesting that its core proteins, e.g., syndecans and glypicans, may provide direct support to the glycocalyx. Other cytoskeletal proteins such as spectrin may also get involved, as it forms a hexagonal arrangement with the actins at the intracellular side of the cell membrane.

Actin depolymerisation was reversible when cells were refreshed with the CD-free medium. The disrupted cytoskeleton started to restore after 10 min and became fully repolymerised within an hour. Rapid actin disruption induced obvious cell retraction and the subsequent repolymerisation facilitated cell respreading. It is worth noting that cell retraction and respreading reflect changes in the cell membrane tension (Gauthier et al. 2012), which has been shown to modulate the development and organisation of membrane-bound molecules (Diz-Munoz et al. 2013; Gauthier et al. 2011). Our results showed that neither the actin depolymerisation and the resulting cell retraction (which leads to a decrease in the cell membrane tension), nor the subsequent actin repolymerisation and cell respreading (which increases the membrane tension) compromised the glycocalyx layer. The cell membrane itself was able to support the glycocalyx layer in static culture medium.

Under steady shear stimulation, actin filaments were observed to relocate preferentially at the cell periphery, reoriented and elongated along the flow direction. This reorganisation led to cell realignment and elongation during 24-h shear stress exposure. The glycocalyx was seen to redistribute towards the downstream region of the cell membrane. The spatial redistribution of the glycocalyx in response to shear stress has been investigated by a number of groups. Yao et al. (2007) reported that the glycocalyx at the end of 24-h stimulation relocated from the central region to the cell periphery in their study using bovine aortic endothelial cells (BAECs) and HUVECs. Zeng and Tarbell (2014) demonstrated that the glycocalyx on rat fat pad endothelial cells and BAECs clustered to the downstream of cell surface at an early stage (${<}30~\hbox {min}$), but was subsequently resorted to a uniform pattern at 24 h. They speculated that this was due to the enhanced biosynthetic activity induced by the shear flow. Different component (e.g., HS or HA) of the glycocalyx was stained in these studies, which may explain the discrepancy described. In our study, we have used the wheat germ agglutinin to target the general saccharides of proteoglycans and glycoproteins of the glycocalyx. There are a number of other factors that may contribute to the observed differences, for example, the thickness of the glycocalyx layer changes with the area and phenotype of blood vessels (Gao and Lipowsky 2010; Haldenby et al. 1994; Savery and Damiano 2008; van Haaren et al. 2003; Yen et al. 2012); different components of the glycocalyx may have very different susceptibility to the shear stress (Arisaka et al. 1995; Gouverneur et al. 2006) and different renewal rates (Giantsos-Adams 2013), which affect the remodelling pattern of the glycocalyx on endothelial cells.

We observed that with persistent actin depolymerisation, the glycocalyx layer on HUVECs and HAECs was both disrupted by the shear stress. The substantial reduction in MFI confirmed the disintegration of the glycocalyx. The loss of the glycocalyx may be attributed to the disturbed mechanotransduction. The glycocalyx serves as a mechanotransducer in response to shear stress, which in turn enables endothelial cells to transit from cobblestone-like morphology to an elongated pattern. This adaptation to flow is through actin reorganisation and is believed to minimise the shear stress on endothelial cell surface (Barbee et al. 1994). Persistent actin depolymerisation weakens the anchoring strength of the core proteins that support the glycocalyx. In other words, the actin cytoskeleton provides a scaffold to stabilise the glycocalyx in response to shear stress. There is another possibility that the actin cytoskeleton contributes to the stability of the glycocalyx via modulation of its dynamic renewal process. The glycocalyx continuously develops and sheds on the cell surface. Giantsos-Adams (2013) recently proposed that the coordination of exocytosis and endocytosis, rather than other steps including de novo production from ribosome and posttranslational modification at Golgi apparatus, determined the differential coverage of HS between static and shear conditions. Given the fact that actin dynamics plays an important role in the regulation of exocytosis and endocytosis (e.g., neurotransmitter from neuron synapse and insulin from pancreatic $\upbeta$-cells) (Porat-Shliom et al. 2013), its reorganisation to shear stress may modulate the membrane trafficking to maintain the glycocalyx on the endothelial cell surface. Current work in our laboratory is probing into this interesting area of research.

It should be pointed out the current study applied laminar shear flow. Further study is needed to clarify the contribution of the actin cytoskeleton to the stability of the glycocalyx under other flow conditions, such as oscillatory and pulsatile flows. Also attention should be paid to the discrepancy between in vivo and in vitro studies. It has been demonstrated that the glycocalyx is much thicker in native HUVECs than in cultured HUVECs (Chappell 2009). Whether the thickening of the glycocalyx could lead to an increased susceptibility to the loss of the cytoskeleton remains to be determined. Nevertheless, the current study provides an insight into the role of the actin cytoskeleton in the stability of the glycocalyx under static and flow conditions in vitro and may lead to new strategies to prevent the glycocalyx shedding in cardiovascular diseases.
